# A Case of Rare Hormone‐Sensitive Primary Adenoid Cystic Carcinoma of the Breast Diagnosed by Preoperative Core Needle Biopsy: A Case Report

**DOI:** 10.1155/cris/2441220

**Published:** 2026-09-15

**Authors:** Keiichi Takahashi

**Affiliations:** ^1^ Takahashi Breast and Gastroenterology Clinic, Osaka, Japan

**Keywords:** adenoid cystic carcinoma, core needle biopsy, hormone-sensitive

## Abstract

Adenoid cystic carcinoma (ACC) of the breast, a special histologic subtype of invasive carcinoma, most commonly originates in the salivary glands and is also found in the cervix, skin, bronchi, kidneys, esophagus, and prostate. ACC originating in the breast is quite rare, accounting for less than 0.1% of all breast cancers. Metastasis to lymph nodes or distant sites is very rare, and the “triple‐negative” phenotype (negativity for hormone receptors and human epidermal growth factor receptor 2 [HER2]) is often noted. However, within the high prevalence of the triple‐negative phenotype, hormone‐sensitive primary ACC of the breast has been extremely rare, with few cases reported. The patient presented with pain around the right nipple persisting for several years. Mammography (MMG) and ultrasonography (US) revealed a mass in the right breast. Core needle biopsy (CNB) was performed. The results demonstrated that atypical cells proliferated invasively, exhibiting mesh‐like, glandular, trabecular, and other structures. Formation of glands with eosinophilic discharge and pseudoglandular cavities due to retention of a mucinous edema‐like matrix was observed, suggesting ACC. Skin‐sparing mastectomy of the right breast and sentinel lymph node biopsy (SSM + SLNB) were performed with simultaneous breast reconstruction by implantation of tissue expanders. The results of surgical pathology were as follows: ACC, pT2 (5.0 cm × 3.8 cm × 2.3 cm), pN0 (sn 0/2), HG2, ly1, v1, estrogen receptor (ER) 20%, progesterone receptor (PgR) <1%, HER2 1+, MIB‐1 index 15%.

## 1. Introduction

Adenoid cystic carcinoma (ACC) of the breast, a special histologic subtype of invasive carcinoma, is rare, accounting for less than 0.1% of all breast cancers [1]. Although the histological appearance is similar to that of primary ACC of the salivary glands, the prognosis is better than that of primary ACC of the salivary glands, and metastasis to lymph nodes or distant sites is very rare. Metastasis to lymph nodes or distant sites is very rare, and the triple‐negative phenotype (negative for hormone receptors and human epidermal growth factor receptor 2 [HER2]) is often noted. However, hormone‐sensitive primary ACC has been extremely rare, with few cases being reported.

As a reason, ACC is believed to originate from the ductal epithelium or myoepithelium [2]. The diagnostic criteria for ACC require a biphasic cellular pattern consisting of myoepithelial and epithelial cells [2]. Histologically, ACC can sometimes resemble invasive ductal carcinoma, which can lead to misdiagnosis in core needle biopsy (CNB) specimens [3]. About 50% of breast ACC cases are misdiagnosed [2]. Since the prognosis of ACC of the breast is far better than that of the same disease occurring in other organs, accurate preoperative histopathological diagnosis is important for appropriate treatment to avoid excessive treatment that causes greater invasion and burden on the body.

## 2. Case Report

A 57‐year‐old woman visited the author’s clinic, citing pain around her right nipple that had persisted for several years as her chief complaint. There is nothing of note in the patient’s medical history or family history. Physical examination, breast ultrasonography (US), and mammography (MMG) were performed.

**Figure 1:**
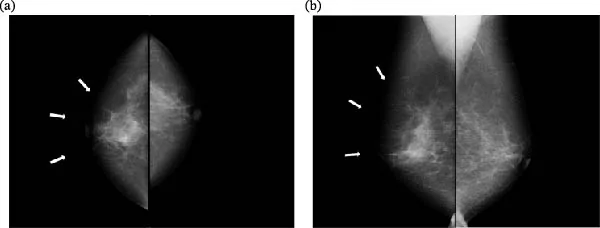
Mammography (a, b). MMG revealed a mass shadow of ~5 cm in the craniocaudal and mediolateral oblique views, localized asymmetric lesions with tumor shadows were observed in the upper‐outer and upper‐inner quadrants of the right breast, respectively, which was diagnosed as a category 5 lesion.

MMG revealed a mass shadow of ~5 cm in the craniocaudal and mediolateral oblique views; localized asymmetric lesions with tumor shadows were observed in the upper‐outer and upper‐inner quadrants of the right breast, respectively, which was diagnosed as a category 5 lesion (Figure 1a,b).

**Figure 2:**
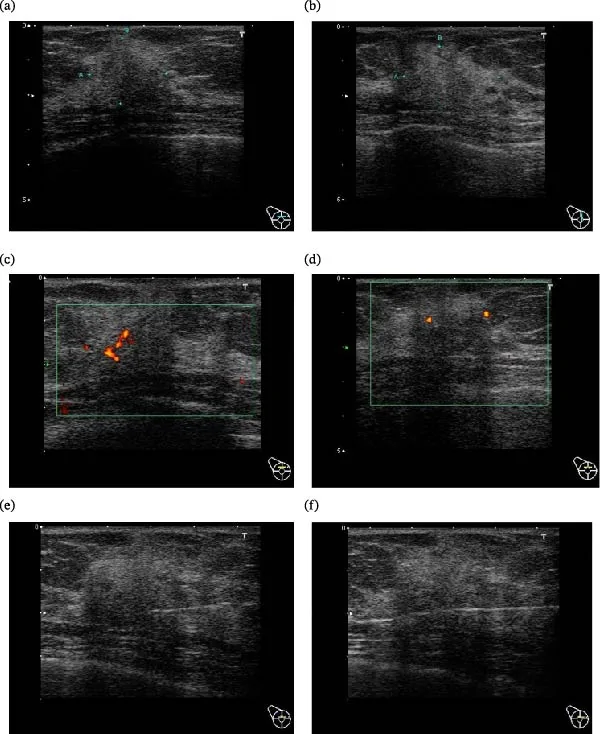
Breast ultrasonography (a, b, c, d, e, f). Breast US revealed a heterogeneous mass measuring ~5 cm with an irregular margin and ill‐defined border in the upper‐outer and upper‐inner quadrants of the right breast, respectively, which was diagnosed as a category 5 lesion (a, b). Color Doppler echo imaging showed a hypervascular pattern (c, d). CNB was performed (e, f).

**Figure 3:**
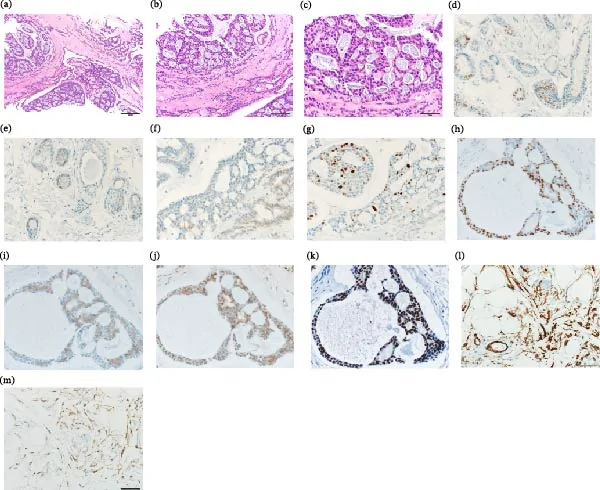
Pathological analysis (a, b, c, d, e, f, g). (a)【H‐E stain, x100】, (b)【H‐E stain, x200】, (c)【H‐E stain, ×400), (d)【ER stain, x400】, (e)【PgR stain, x400】, (f)【HER2 stain, x400】, (g)【MIB‐1 index stain, x400】, (h)【p63 stain, x400】, (i)【CD117 stain, x400】, (j)【S‐100 stain, x400】, (k)【SOX10 stain, x400】, (l)【CD34 stain, x400】, (m)【CD10 stain, x400】. The pathological findings of the CNB specimen indicated that atypical cells proliferated invasively, exhibiting mesh‐like, glandular, trabecular, and other structures. Formation of glands with eosinophilic discharge and pseudoglandular cavities due to retention of mucinous edema‐like matrix was observed, suggesting adenoid cystic carcinoma (ACC). Immunohistochemical (IHC) staining of estrogen receptor (ER) showed positivity in 20% of the tumor cells (d). IHC staining of the progesterone receptor (PgR) receptor showed positivity in less than 1% of tumor cells (e). Human epidermal growth factor receptor 2 (HER2) IHC staining showed score 1+ positivity in the tumor cells (f). IHC staining of the MIB‐1 index showed positivity in 15% of tumor cells (g). IHC staining of the p63, which is considered specific for the diagnosis of ACC showed positivity in the myoepithelial cells of the tumor (h). IHC staining of the CD117: while this marker is known to show positivity in normal mammary epithelial cells, negativity in invasive ductal carcinoma, and strongly positivity in ACC, it showed highly positivity in this case (i). IHC staining of the S‐100: while this marker is known to show positivity in the myoepithelial cells of the tumor, it showed highly positivity in this case (j). IHC staining of the SOX10, which is considered highly sensitive diagnostic marker for ACC showed 100% diffuse nuclear positivity in both the luminal and basal layers of the tumor (k). IHC staining of the CD34: spindle cells are one of the cellular components constituting the tumor, along with adipocytes and thick collagen fibers; in this case, the spindle cells showed strong positivity (l). IHC staining of the CD10 showed positivity in the spindle cells of the tumor component (m).

Breast US revealed a heterogeneous mass measuring ~5 cm with an irregular margin and ill‐defined border in the upper‐outer and upper‐inner quadrants of the right breast, respectively, which was diagnosed as a category 5 lesion (Figure 2a,b). Color Doppler echo imaging showed a hypervascular pattern (Figure 2c,d). CNB was performed, as shown in Figure 2e,f. The results demonstrated that atypical cells proliferated invasively, exhibiting mesh‐like, glandular, trabecular, and other structures. Formation of glands with eosinophilic discharge and pseudoglandular cavities due to retention of mucinous edema‐like matrix was observed, suggesting ACC (Figure 3a–c). Immunohistochemical (IHC) staining of the estrogen receptor (ER) showed positivity in 20% of the tumor cells (Figure 3d). IHC staining of the progesterone receptor (PgR) showed positivity in less than 1% of tumor cells (Figure 3e). IHC staining of the HER2 IHC staining showed score 1+ positivity in the tumor cells (Figure 3f). IHC staining of the MIB‐1 index showed positivity in 15% of tumor cells (Figure 3g). IHC staining the p63, which is considered specific for the diagnosis of ACC, showed positivity in the myoepithelial cells of the tumor (Figure 3h).

IHC staining of CD117: while this marker is known to show positivity in normal mammary epithelial cells, negativity in invasive ductal carcinoma, and strong positivity in ACC, it showed high positivity in this case (Figure 3i).

IHC staining of the S‐100: while this marker is known to show positivity in the myoepithelial cells of the tumor, it showed high positivity in this case (Figure 3j).

IHC staining of SOX10, which is considered a highly sensitive diagnostic marker for ACC, showed 100% diffuse nuclear positivity in both the luminal and basal layers of the tumor (Figure 3k).

IHC staining of the CD34: spindle cells are one of the cellular components constituting the tumor, along with adipocytes and thick collagen fibers; in this case, the spindle cells showed strong positivity (Figure 3l).

IHC staining of CD10 showed positivity in the spindle cells of the tumor component (Figure 3m).

Based on the above findings, the tumor cells exhibit a biphasic pattern: the luminal epithelial cells showed CD117 positivity, while the myoepithelial cells on the extraluminal side showed p63‐ and S‐100‐positivity.

**Figure 4:**
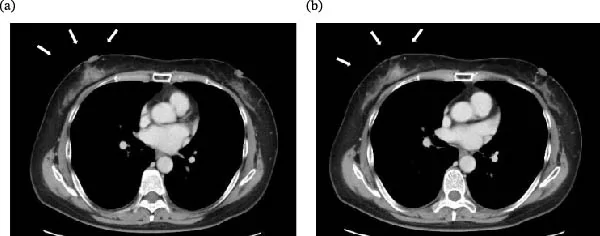
Computed tomography (a, b). Contrast‐enhanced computed tomography (CT) showed an area of ill‐defined borders and segmental faint enhancement immediately in the upper‐outer and upper‐inner quadrants of the right breast, corresponding to breast cancer. No lymph node or distant metastasis was observed.

**Figure 5:**
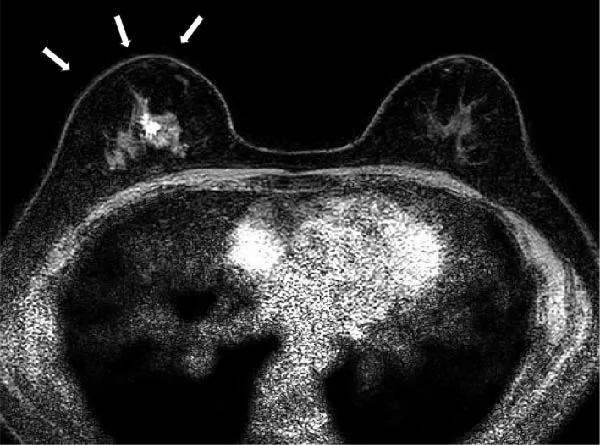
Magnetic resonance imaging. Contrast‐enhanced magnetic resonance imaging (MRI) of the breast showed an irregular mass in the upper‐outer and upper‐inner quadrants of the right breast, consistent with malignancy, and no axillary lymph node metastasis.

Contrast‐enhanced CT identified an area of ill‐defined borders and segmental faint enhancement immediately in the upper‐outer and upper‐inner quadrants of the right breast, corresponding to breast cancer. No lymph node or distant metastasis was observed (Figure 4a,b). Contrast‐enhanced MRI of the breast showed an irregular mass in the upper‐outer and upper‐inner quadrants of the right breast, consistent with malignancy, and no axillary lymph node metastasis (Figure 5). Bone scintigraphy detected no bone metastases. Skin‐sparing mastectomy of the right breast and sentinel lymph node biopsy (SSM + SLNB) were performed with simultaneous breast reconstruction by the implantation of tissue expanders. The results of surgical pathology were as follows: ACC, pT2 (5.0 cm × 3.8 cm × 2.3 cm), pN0 (sn 0/2), HG2, ly1, v1, ER 20%, PgR less than 1%, HER2 1+, and MIB‐1 index 15%.

## 3. Discussion

ACC most commonly originates in the salivary glands and is also found in the cervix, skin, bronchi, kidneys, esophagus, and prostate [1, 4]. However, its occurrence in the breast is extremely rare and accounts for less than 0.1% of all breast cancers [1, 2, 4]. The age of onset ranges widely from 19 to 97 years, with a peak at 50–60 years. It has been reported that the average diameter of the tumor is 2–3 cm, and the maximum diameter observed so far is 15 cm [5]. While ACC is generally classified as TNBC, the frequency of axillary lymph node metastasis is low (0%–2%) and distant metastasis is rare, resulting in a favorable prognosis [2, 3]. It is characterized by a very good 10‐year survival rate of ~95% [2].

Primary ACC of the breast is usually identified by a subareolar mass or breast pain. It has been reported that such pain is associated with the perineural invasion of tumor cells and contraction of myoepithelial cells [6]. Although the histological picture is similar to that of primary ACC of the salivary gland, the prognosis is better than that of the salivary gland counterpart, and distant metastasis or recurrence is rare. Despite its triple‐negative phenotype—i.e., negativity for ER, PgR, and HER2—primary ACC of the breast has a better prognosis than other histological types of breast cancer, with very rare local recurrence and distant metastasis [3, 7].

The diagnostic criteria for ACC require a biphasic cellular pattern consisting of myoepithelial and epithelial cells. Tumor cells are distributed around small cysts and form both true glands and pseudoglandular spaces containing an eosinophilic basement membrane‐like material and basophilic mucin. Subtypes include the tubular, cribriform, and solid types, which may be present alone or in combination [8, 9].

1. In the tubular type, the inner layer consists of epithelial cells, surrounded by myoepithelial cells; glandular spaces or pseudoglandular lumens are noted.
2. In the cribriform type, myoepithelial cells line pseudoglandular lumens, with epithelial cells interposed between them.
3. In the solid type, the image shows proliferation primarily consisting of myoepithelial cells.

In this case, it was confirmed that p63‐ and S‐100‐positive myoepithelial cells were shown to surround clusters of CD117‐positive epithelial cells, indicating a tubular pattern.

Regarding the operative procedure, the distant metastasis rate after partial mastectomy does not differ from that after total mastectomy. Radiation to the remaining mammary gland reportedly contributes to a reduction of recurrence in the remaining mammary gland in ACC as well.

While the lesion often appears relatively localized on imaging, it often spreads invasively to surrounding tissues on histopathological images. There are few imaging findings characteristic of ACC of the breast. ACC of the breast usually exhibits a low‐grade, low‐proliferative subtype, with less than 2% of cases showing axillary metastasis and less than 2% of cases showing distant metastasis [10, 11]. Metastasis from ACC is rare, but when it occurs, the lungs are most commonly affected, followed by the liver, bones, and kidneys. This is consistent with the metastatic sites of salivary gland ACC. Extremely rare metastasis to the brain has been reported. Since lymph node or distant metastasis is rare, the prognosis is very good. However, there are reports indicating that treatment with local excision alone can disadvantageously increase the risk of local recurrence [3]. Since the rate of metastasis to the axillary lymph nodes is low, excision does not bring a beneficial role in the treatment of ACC. If the diameter of the breast tumor is 3 cm or larger in the case of high‐grade malignancy, SLNB is a prudent option [5, 10]. At present, axillary lymph node dissection (ALND) is not recommended for ACC of the breast. Hormone sensitivity is very low, and the triple‐negative phenotype has been noted in most cases. Few hormone‐sensitive cases have been reported. While the prognosis of triple‐negative breast cancer is generally considered poor, a good prognosis for ACC of the breast is achievable by surgical resection alone despite its triple‐negative phenotype. As a reason, ACC is believed to originate from the ductal epithelium or myoepithelium [2]. The diagnostic criteria for ACC require a biphasic cellular pattern consisting of myoepithelial and epithelial cells [2]. Histologically, ACC can sometimes resemble invasive ductal carcinoma, which can lead to misdiagnosis in CNB specimens [3]. About 50% of breast ACC cases are misdiagnosed [2].

Because the prognosis for ACC of the breast is far more favorable than for the same disease occurring in other organs, an accurate preoperative histopathological diagnosis is essential for appropriate treatment in order to avoid excessive treatment that would increase the physical invasiveness and burden on the patient.

## 4. Conclusion

A case of very rare hormone‐sensitive primary ACC of the breast was encountered.

Because the prognosis for ACC of the breast is far more favorable than that for the same disease occurring in other organs or for typical invasive ductal carcinoma, an accurate preoperative histopathological diagnosis is crucial for providing appropriate treatment.

**Nomenclature**
ACC: Adenoid cystic carcinoma
CNB: Core needle biopsy
MMG: Mammography
US: Ultrasonography
IHC: Immunohistochemical.

## Author Contributions

The author has nothing to report.

## Funding

This research did not receive any specific grant from funding agencies in the public, commercial, or not‐for‐profit sectors.

## Ethics Statement

This work does not require ethical considerations or approval.

## Consent

Informed consent was obtained from the patient to participate in the study and for the publication of this case report and any accompanying images.

## Conflicts of Interest

The author declares no conflicts of interest.

## Data Availability

The data used to support the findings of this study are available from the corresponding author upon reasonable request.
